# Neoadjuvant and adjuvant systemic therapy in HCC: Current status and the future

**DOI:** 10.1097/HC9.0000000000000430

**Published:** 2024-06-03

**Authors:** Amit G. Singal, Mark Yarchoan, Adam Yopp, Gonzalo Sapisochin, David J. Pinato, Anjana Pillai

**Affiliations:** 1 Department of Medicine, UT Southwestern Medical Center, Dallas, Texas, USA; 2 Sidney Kimmel Comprehensive Cancer Center, Johns Hopkins University School of Medicine, Baltimore, Maryland, USA; 3 Department of Surgery, UT Southwestern Medical Center, Dallas, Texas, USA; 4 Department of Surgery, University of Toronto and University Health Network, Toronto, Ontario, Canada; 5 Department of Surgery and Cancer, Faculty of Medicine, Imperial College London, Hammersmith Hospital, Du Cane Road, London, UK; 6 Department of Translational Medicine, Division of Oncology, University of Piemonte Orientale, Novara, Italy; 7 Department of Medicine, University of Chicago, Chicago, Illinois, USA

## Abstract

Surgical therapies in patients with early-stage HCC can afford long-term survival but are often limited by the continued risk of recurrence, underscoring an interest in (neo)adjuvant strategies. Prior attempts at adjuvant therapy using tyrosine kinase inhibitors failed to yield significant improvements in recurrence-free survival or overall survival. Advances in the efficacy of systemic therapy options, including the introduction of immune checkpoint inhibitors, have fueled renewed interest in this area. Indeed, the IMBrave050 trial recently demonstrated significant improvements in recurrence-free survival with 1 year of adjuvant atezolizumab plus bevacizumab in high-risk patients undergoing surgical resection or ablation, with several other ongoing trials in this space. There is a strong rationale for consideration of the administration of these therapies in the neoadjuvant setting, supported by early clinical data demonstrating high rates of objective responses, although larger trials examining downstream outcomes are necessary, particularly considering the possible risks of this strategy. In parallel, there has been increased interest in using systemic therapies as a bridging or downstaging strategy for liver transplantation. Current data suggest the short-term safety of this approach, with acceptable rates of rejection, so immunotherapy is not considered a contraindication to transplant; however, larger studies are needed to evaluate the incremental value of this approach over locoregional therapy. Conversely, the use of immunotherapy is currently discouraged after liver transplantation, given the high risk of graft rejection and death. The increasing complexity of HCC management and increased consideration of (neo)adjuvant strategies highlight the critical role of multidisciplinary care when making these decisions.

## INTRODUCTION

HCC is the fourth leading cause of cancer-related mortality worldwide and a leading cause of death in patients with cirrhosis.^1^ The prognosis of patients with HCC differs by tumor stage, highlighting the importance of early detection.^2^ Patients with advanced HCC have a median survival of 1–3 years, whereas surgical therapies, including liver transplantation (LT)and surgical resection, facilitate median survival beyond 5 years in patients with early-stage HCC.^3^ Although these therapies are curative in intent, patients remain at high risk of recurrence.^4^ Patients with recurrence have significantly worse survival than those who remain disease-free.^4^ (Neo)adjuvant therapy has improved postsurgical outcomes for many cancers such as pancreas,^5^ breast,^6^ lung,^7^ and colorectal cancer^8^; however, this has not been the case in HCC to date, partly related to limited systemic therapy options, safety, and efficacy.

Until 2020, first-line systemic therapies for HCC were limited to 2 tyrosine kinase inhibitors, sorafenib and lenvatinib.^9^,^10^ Although both improved survival in the advanced stage setting, median survival for both was ~12–13 months in their respective registration randomized clinical trials, and neither induced durable responses in a high proportion of patients. When tested in an adjuvant setting in the STORM Trial, sorafenib failed to improve RFS compared to placebo.^11^

Since 2019, there has been marked progress in the systemic therapy landscape for HCC.^12^ The combination of atezolizumab plus bevacizumab was the first systemic therapy to show superior overall survival (OS) to sorafenib in the IMBrave150 Trial, demonstrating a median survival of 19 months and objective responses in 30% of the patients, compared to 13.4 months and 11%, respectively, for sorafenib.^13^ Soon after, the combination of durvalumab and tremelimumab also showed superior survival compared to sorafenib in the HIMALAYA Trial, with a median survival of 16 months and 25% of the patients living beyond 4 years.^14^ Consequently, there has been marked interest in evaluating these therapies in earlier stages of the disease, including trials of systemic therapy versus locoregional therapy in “transarterial chemoembolization (TACE) unsuitable” patients, combinations of systemic therapy with embolic or radiation-based therapies in intermediate-stage HCC, and as (neo)adjuvant therapy with surgical therapies in early-stage disease. Indeed, the IMBrave050 Trial recently reported a significant improvement in RFS with atezolizumab plus bevacizumab in patients at high risk of recurrence after surgical resection or local ablation.^15^

Herein, we discuss the rationale, existing data, ongoing trials, and considerations regarding the use of systemic therapy in the (neo)adjuvant setting for patients with HCC.

### Treatment considerations for patients undergoing surgical resection

#### Current patient selection and anticipated outcomes

Clinical practice guidelines recommend surgical resection as a preferred treatment option in patients with early-stage HCC and preserved liver function in the absence of clinically significant portal hypertension.^3^,^16^,^17^ Surgical resection in early-stage HCC affords 5-year OS rates approaching 70%^18^,^19^; however, recurrence occurs in 50%–70% of patients at 5 years (Table 1). There is a bimodal pattern of early (within 2 years of resection) and late (after 2 years) recurrence.^4^ Early recurrence is more likely due to occult intrahepatic metastases from the resected tumor, whereas recurrences after 2 years, based on studies of clonal origin, are typically related to de novo tumors.^27^

**TABLE 1:** Selected studies of clinical outcomes after surgical resection

References	Study period	Number of patients	Proportion with recurrence, %	Median time to recurrence (mo)	Five-year disease-free survival, %	Five-year overall survival, %
Tabrizian^4^	1988–2011	661	54	22	N/A	45
Tsilimigras^20^	2005–2017	756	BCLC A 46 BCLC B/C 60	NR	BCLC A 27 BCLC B/C 22	BCLC A 77 BCLC B/C 52
Shah^21^	1992–2004	193	51	34	39	53
Chapman^22^	1990–2011	884	NR	NR	30	53
Han^23^	1996–2007	610	49	N/A	46	64
Huang^24^	2000–2008	1313	NR	15.0	32	46
Yoh^25^	1995–2014	989	69	12.1	NR	47
Shindoh^26^	1995–2021	1616	61	16.3	36	78

Abbreviation: BCLC, Barcelona Clinic Liver Cancer.

The appropriate selection of patients eligible for surgical resection requires a multilevel consideration of tumor-level and patient-level factors balancing tumor biology and concomitant liver dysfunction. Although perioperative mortality following HCC resection has decreased from 7% in the 1990s to below 3% in recent years,^28^ careful patient selection remains essential to ensure that oncological benefits outweigh the potential for post-hepatectomy liver failure.^29^,^30^ Surgical resection is generally reserved for patients with Child-Turcotte-Pugh A cirrhosis and an adequate post-resection future liver remnant (typically > 40% in cirrhotic livers) with an absence of clinically significant portal hypertension.^17^,^31^ Surrogates of absent clinically significant portal hypertension, including platelet count >10,000/mL, ascites, and portosystemic varices, are commonly used measures due to the invasive nature of measuring HVPG; however, mild portal hypertension (eg, platelet count >90,000/mL) may be tolerated with minor resections, particularly if performed using a minimally invasive approach.

While these considerations have remained largely unchanged, some experts have proposed expanded indications for HCC surgical resection based on tumor-related factors. Some centers, particularly in Asia, have moved away from guideline criteria limiting resection to solitary tumors to resection of multifocal tumors and/or with the presence of macrovascular tumor invasion in carefully selected patients. In patients presenting with multifocal HCC within the Milan criteria, surgical resection can yield 5-year OS rates ranging from 49% to 69%.^32^,^33^ These favorable rates compared to 3-year survival rates of 26%–29% following locoregional treatment (TACE or transarterial radioembolization) have led many non-Western centers to adopt surgical resection as the preferred treatment modality in patients presenting with multifocal HCC who are not transplant candidates.^34^,^35^ A meta-analysis of 18 studies comparing surgical resection with TACE reported a significant survival advantage for surgical resection among patients with Barcelona Clinic Liver Cancer stage B HCC (HR, 0.56; 95% CI, 0.35–0.90).^36^ In patients presenting with multifocal HCC tumors outside of Milan criteria, the outcomes are not as favorable as 5-year OS rates range from 12% to 24%. However, recent studies have demonstrated that surgical resection may provide improved survival compared to locoregional therapy, even in this expanded indication cohort.^36^,^37^

HCC has a known proclivity for macrovascular invasion and presents most frequently as a portal vein tumor thrombus (PVTT). Resection of tumors with associated PVTT is controversial due to the high recurrence rates due to the vascular spread of metastatic disease and is typically recommended to undergo systemic therapy. However, the paradigm of not offering surgical resection in this cohort of patients has recently been challenged in high-volume centers worldwide, resulting in both low perioperative mortality and favorable oncological outcomes. The overall goal of hepatic resection for HCC with concomitant PVTT includes not only a margin-negative resection but also tumor extirpation within the portal system to mitigate further clinical manifestation of portal hypertension and hematogenous tumor dissemination. In patients presenting with HCC and PVTT limited to second-order or segmental portal branches or more distally (Liver Study Group of Japan Vp1 or Vp2), en bloc surgical resection was associated with a median OS of 73.4 months (Vp1) and 38.0 months (Vp2). In the same nationwide series, the median OS in patients receiving systemic therapy alone was 16.2 months and 8.2 months for Vp1 and Vp2 PVTT, respectively.^38^ Several retrospective studies, including patients with HCC and concomitant Vp3 (involving first-order branches) PVTT, demonstrate a benefit from surgical resection in the presence of preserved liver function.^39^,^40^ Given the high recurrence rate and increased perioperative mortality, scoring systems have been described to select patients who may benefit the most from surgical resection with concomitant PVTT.^39^,^40^ These systems and other retrospective studies have demonstrated high rates of recurrence and perioperative mortality in Vp4 PVTT resections; therefore, hepatic resection should only be considered in select patients with Vp1-Vp3 PVTT.

With significant improvements in systemic therapy regimens for advanced-stage HCC coupled with recent reports detailing (neo)adjuvant trials in early-stage HCC, it remains unclear if the role of surgical resection in HCC will expand in the future. However, until future studies reach completion, surgical resection in HCC for expanded indications should only be performed in experienced centers and only after careful patient selection.

#### Rationale for adjuvant therapy

The remarkable advances in the use of immune checkpoint inhibitors (ICI) in advanced HCC, with evidence of measurable and durable responses in up to 30% of patients, have rapidly led to a growing interest in the integration of systemic therapy in the management of earlier-stage HCC.

The potential for adjuvant use of systemic anticancer therapy as a strategy to reduce the risk of relapse after definitive treatment of cancer dates to 1958.^41^ The recognized potential for cytotoxic chemotherapy to eradicate dormant and unmeasurable disseminated disease led to the approval of several cytotoxic regimens across major tumor sites, including breast, lung, and colorectal cancer, and many others. Patient selection for adjuvant therapy has been refined over the years by identification of oncological features associated with worse prognosis (eg, larger tumor size, poor differentiation, positive resection margins) or certain molecular subtypes (eg, microsatellite unstable colorectal cancer or hormone receptor positivity in breast cancer).

HCC stands in stark contrast to other tumor types with a historic lack of proven adjuvant therapy despite known risk factors for early recurrence, including higher tumor burden, elevated alpha fetoprotein levels, microvascular invasion, and poor tumor differentiation. Further, the proven relationship between programmed cell death protein ligand-1 (PD-L1) expression and, more broadly, an immune-exhausted tumor microenvironment and adverse outcomes after surgery highlight a strong rationale for adjuvant ICI therapy. Advantages of postsurgical immunotherapy include confirmation of complete response to surgical therapy and risk stratification based on histopathologic analysis. Adjuvant therapy does not oblige patients to defer primary therapy and leaves ample room (up to 16–18 wks in adjuvant trials across indications) for postsurgical recovery ahead of systemic therapy initiation.

#### Potential outcomes for efficacy of (neo)adjuvant therapy

The goal of systemic anticancer therapy in early-stage HCC is to increase the chances of cure. With relapse being tightly linked with survival, it is expected that a systemic therapeutic strategy that positively impacts the risk of recurrence would lead to an improvement in OS. However, the broadening armamentarium of surgical, locoregional, radiation, and systemic therapies currently available to patients with relapsed HCC makes it difficult for the relationship between adjuvant therapy exposure and OS to emerge consistently across studies. This is particularly true given OS is highly sensitive to crossover.

RFS and time to recurrence have been used as end points to measure incremental improvement from adjuvant immunotherapy exposure to oncological outcomes.^42^ The 2 end points are not interchangeable, as they measure the interval between initiation of adjuvant treatment and recurrence with RFS or without time to recurrence considering death as a meaningful event. RFS is traditionally preferred as an end point for adjuvant therapy, although deaths unrelated to oncological disease progression might affect the surrogacy of RFS as a measure of the success of adjuvant therapy.

#### Data for adjuvant therapy

Adjuvant therapy for patients with HCC to improve RFS has been an unmet need for years. Antiviral agents remain the only therapy recommended in this setting until recently given improved OS, albeit without effect on the risk of tumor recurrence.^43^,^44^ Several large clinical trials failed to demonstrate a benefit with agents including retinoids, interferon, and vitamin K.^45^,^46^,^47^,^48^ Similarly, the STORM trial failed to demonstrate improved RFS with adjuvant sorafenib in patients with HCC undergoing resection or thermal ablation compared to placebo (HR, 0.94; 95% CI: 0.78–1.13).^11^ A trial from China suggested improved RFS with the use of adjuvant intra-arterial chemotherapy, although these results still require validation in patient populations in Western societies without HBV.^49^

The open-label phase III RCT comparing atezolizumab plus bevacizumab versus active surveillance (IMbrave050) in the adjuvant setting for patients with HCC at high risk of recurrence after resection or local ablation was the first to demonstrate positive results.^15^ High-risk features for resection patients included 1–3 tumors with a size > 5 cm, more than 3 tumors each ≤ 5 cm, and vascular invasion or poor tumor differentiation in those with 1–3 tumors each ≤ 5 cm. High-risk features for ablation included a unifocal tumor > 2 cm but ≤5 cm or 2–4 tumors each ≤ 5 cm. The trial included 668 patients who were randomized to active surveillance or atezolizumab plus bevacizumab within 12 weeks of the surgery. Patients were treated for 12 months unless the patient experienced disease recurrence or dose-limiting toxicity. Most patients in the trial (88%) had undergone surgical resection, with 90% having a unifocal tumor and a median tumor size of 5.5 cm. Most patients in the ablation group also had a unifocal tumor, with a median tumor size of 2.5 cm. After a median follow-up of 17.4 months, the trial achieved its primary end point for the superiority of RFS (RFS, 0.72, 95% CI: 0.56–0.93), with 12-month RFS estimates of 78% versus 65% for the intervention and surveillance arms, respectively. The curves appear to potentially converge around month 21, although it is unclear if this is related to censoring with a short duration of follow-up or related to the combination delaying instead of preventing recurrence.

The median duration of treatment for atezolizumab plus bevacizumab was 11 months, with 34.9% of patients experiencing grade 3–4 treatment-related adverse events. Immune-related adverse events requiring steroids were observed in 8.4% of the patients treated with atezolizumab plus bevacizumab, and 8.7% discontinued both agents due to an adverse event. Data evaluating OS (a secondary endpoint) were immature at the interim analysis, and continued follow-up is ongoing.

There are several ongoing trials evaluating other ICIs in the adjuvant setting (Table 2). In brief, nivolumab monotherapy and pembrolizumab monotherapy are being evaluated in the Checkmate-9DX and Keynote-937 Trials, respectively. The combination of adjuvant durvalumab with or without bevacizumab is also being evaluated in the EMERALD-2 Trial. This latter trial is of interest, as it will inform the need for bevacizumab versus ICI alone in the adjuvant setting. Each of these trials has completed enrollment, and we are now awaiting results.

**TABLE 2:** Select ongoing phase III trials evaluating immunotherapy in an adjuvant setting or combined with locoregional therapy

Trial name	Therapeutic agents	Primary outcome	Status
Adjuvant setting			
** **IMBrave050 NCT04102098	Atezolizumab + bevacizumab vs. placebo	RFS	Met primary end point
** **Keynote-937 NCT03867084	Pembrolizumab vs. placebo	RFS and OS	Active, not recruiting
** **Checkmate-9DX NCT03383458	Nivolumab vs. placebo	RFS	Active, not recruiting
** **EMERALD-2 NCT03847428	Durvalumab +/− bevacizumab vs. placebo	RFS	Active, not recruiting
** **JUPITER-04 NCT03859128	Toripalimab vs. placebo	RFS	Active, not recruiting
Combined with locoregional therapy			
** **EMERALD-1 NCT03778957	Durvalumab +/− bevacizumab + TACE vs. TACE alone	PFS	Met primary end point
** **LEAP-012 NCT04246177	Pembrolizumab + lenvatinib + TACE vs. TACE alone	PFS and OS	Active, not recruiting
** **Checkmate-74W NCT04340193	Nivolumab + Ipilimumab + TACE vs. TACE alone	TTP and OS	Active, not recruiting
** **TACE-3 NCT04268888	Nivolumab + TACE vs. TACE alone	TTP and OS	Recruiting
** **EMERALD-3 NCT05301842	Durvalumab + tremelimumab + TACE +/− lenvatinib vs. TACE alone	PFS	Recruiting

Abbreviations: OS, overall survival; RFS, recurrence-free survival; TACE, transarterial chemoembolization; TTP, time to progression.

#### Rationale for neoadjuvant therapy

**FIGURE 1:**
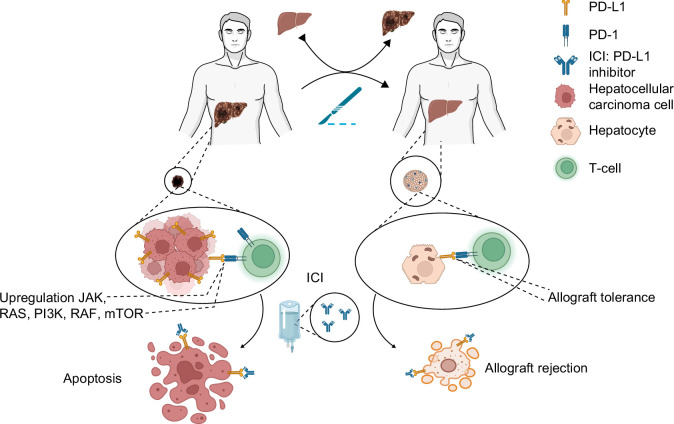
Rationale for immunotherapy in adjuvant versus neoadjuvant setting for patients undergoing surgical resection. There are several benefits (green boxes) and limitations (red boxes) when considering a neoadjuvant approach (left side) versus an adjuvant approach (right side) to improve disease-free and overall survival in patients undergoing surgical resection. Potential end points for (neo)adjuvant trials are described at the bottom of the figure. Abbreviations: MoA, mechanism of action; PD-1, Programmed cell death protein-1; PD-L1, Programmed cell death protein ligand-1; TME, tumor microenvironment.

With early signals of efficacy for immunotherapy in the adjuvant setting, there has been increasing interest in moving ICI therapy into the neoadjuvant setting (Figure 1).^50^ Preclinical evidence accumulated across various malignancies supports the rationale for delivering immunotherapy before definitive surgical treatment of cancer as a therapeutic strategy characterized by superior efficacy and stronger immune cell engagement as opposed to postoperative therapy.^51^,^52^,^53^ It is posited that the presence of an active and measurable tumor might lead to improved neoantigen recognition, DC priming, and ultimately immunological clearance of active and micrometastatic disease. While sensitivity to neoadjuvant ICI varies across histologies, the clinical benefits of adopting preoperative checkpoint inhibitor therapy are manifold. Improved surgical outcomes by primary downsizing/downstaging of the disease may lead to less demolitive surgery and shorter recovery times. Neoadjuvant immunotherapy holds the advantage of inducing antitumor immune reconstitution in a subset of patients with early-stage disease when a more limited tumor burden and lower degree of immune exhaustion could lead to improved and more durable responses compared to advanced disease. Earlier treatment of micrometastatic disease is an equally important aim, given that the persistence of neoplastic clones after treatment is thought to be a key determinant of disease relapse after curative treatment. Neoadjuvant studies are associated with a unique opportunity to derive information on dynamic changes stemming from ICI exposure: a point of greater consequence in HCC where no biomarker of therapeutic benefit from immunotherapy has been solidly established.

Paradigmatic examples highlighting the disruptive effect of neoadjuvant immunotherapy have been demonstrated by the utilization of the anti-PD1 monoclonal antibody dostarlimab in patients who are early-stage mismatch-repair–deficient presenting with rectal cancer, where checkpoint blockade in this patient population was able to lead to complete tumor response in all 12 patients treated, leading to sparing of chemoradiotherapy or surgery.^54^ The benefits of preoperative delivery of checkpoint inhibitor therapy are not exclusive to mismatch-repair deficient tumors but are preserved in oncological indications that are comparatively less sensitive to ICI. In patients with resectable stage IIIb/IVc malignant melanoma, administration of 3 doses of pembrolizumab before surgery followed by 15 cycles of adjuvant therapy led to a significant event-free survival advantage compared to the delivery of a full schedule of 18 cycles of adjuvant treatment.

Neoadjuvant therapy is not without potential downsides. A persistent concern is that patients may progress during neoadjuvant therapy, precluding surgical resection in patients who, at the time of treatment initiation, would have been potentially curable. Whether such patients with rapidly progressive tumors would have benefitted from upfront resection, or conversely, whether testing biology with neoadjuvant immunotherapy instead prevents a potentially morbid surgery in a patient with poor HCC biology who would have inevitably recurred is unknown. The use of neoadjuvant immunotherapy-based combinations could result in treatment toxicities such as serious immune-related adverse events that result in unacceptable surgical delays or preclude surgery. Published studies of neoadjuvant therapy, including anti-PD1 immunotherapy alone or in various combinations, collectively show a low rate of progression events and a low rate of surgical delays due to toxicity. Among 4 recently reported neoadjuvant studies (including series from Johns Hopkins,^55^ Mt. Sinai,^56^ MD Anderson,^57^ and Imperial College London^58^), there were a total of 6 (7%) progression events among 86 treated patients, and no patients missed an opportunity for surgical resection due to adverse events. However, these initial neoadjuvant studies were conducted by centers with high levels of immunotherapy and HCC expertise, and broader studies of neoadjuvant therapy are still needed.

Finally, as systemic therapy moves into the perioperative setting, treatment of disease recurrence may be an emerging challenge. In theory, patients with recurrent HCC following (neo)adjuvant immunotherapy may have acquired resistance by means of immune-resistant clones or have had disease with primary PD1 resistance. A theoretical concern with perioperative immunotherapy is that it may promote the emergence of immune resistance in ways that are different than in the metastatic setting, reducing the total benefit of systemic therapy, although available clinical data do not support this concern. There are no high-quality HCC data to guide treatment decisions for patients who recur following exposure to perioperative immunotherapy. A recent phase III trial examined the role of re-treatment with an ICI by investigating the effect of adding atezolizumab to cabozantinib in patients with disease progression on a prior ICI. In this study, the addition of atezolizumab did not provide any benefit with regard to objective response rates, progression free survival, or OS, but did increase toxicity.^59^ This study, while in a very different treatment context, suggests no role for rechallenging with a PD-L1 inhibitor in the setting of known resistance to anti-PD-L1 or anti-PD1 therapy. Conversely, a small retrospective study reported the clinical benefit of anti-cytotoxic T-lymphocyte associated protein 4 (CTLA4)/PD(L)1 in patients who experienced progression on PD-(L)1 with or without anti-VEGF regimens in HCC.^60^ Some guidelines, including those from the American Association for the Study of Liver Diseases (AASLD), recommend moving on to second-line therapy options (eg, TKIs or anti-CTLA4/PD(L)1 therapy) in patients who experience disease recurrence soon after surgery with perioperative atezolizumab plus bevacizumab.^17^

#### Early data for neoadjuvant therapy

The safety and feasibility of neoadjuvant immunotherapy were recently demonstrated in 4 separate single-institution studies,^55^,^56^,^57^,^58^ which all treated patients with PD1-based regimens for 2–3 months before surgical resection. These initial studies demonstrated pathological response rates of 20%–33% for PD1+tyrosine kinase inhibitor (TKI) (nivolumab plus cabozantinib), PD1 alone (nivolumab and cemiplimab), and PD1+CTLA4 (nivolumab plus ipilimumab), and radiographic objective response rates in up to ~25% of the patients. These studies also collectively demonstrate that objective response rates may underestimate the extent of tumor necrosis at early time intervals. For example, in the neoadjuvant trial of nivolumab plus cabozantinib, among 5 patients achieving pathological responses, only 1 achieved a radiographic response.^55^ Another major conclusion of these studies is that neoadjuvant immunotherapy can modulate the tumor immune microenvironment and can induce a brisk immune infiltrate and tertiary lymphoid structures within patients who achieve a pathological response, consistent with the proposed mechanism of action of neoadjuvant immunotherapy. Among these studies, the clinical trial of nivolumab plus cabozantinib was somewhat unique in its inclusion of patients outside of traditional resection criteria, including patients with major blood vessel involvement or multifocal disease. Even in this group of patients, those achieving pathological responses had outstanding outcomes, suggesting that neoadjuvant immunotherapy may eventually expand the pool of patients achieving successful surgical resection beyond the Barcelona Clinic Liver Cancer A category.^3^

Pathological response has traditionally been used as a clinical end point for studies of neoadjuvant therapy and may potentially serve as a surrogate end point for more clinically relevant end points (eg, RFS, OS). Intuitively, if the primary tumor demonstrates a pathologic response, it follows that the treatment successfully induced systemic antitumor immunity and likely reduced the micrometastatic disease burden, thus delaying or preventing subsequent recurrence. Pathological response was evaluated as a primary end point (along with RFS) in the first pivotal studies of neoadjuvant immunotherapy in triple-negative breast cancer and nonsmall cell lung cancer,^61^,^62^ contributing to Food and Drug Administration approval in both indications. However, whether pathological response is a valid surrogate for more clinically relevant outcomes in HCC is not clearly established. In published studies of neoadjuvant immunotherapy,^55^,^56^,^57^,^58^ patients achieving pathological responses have achieved outstanding clinical outcomes. Indeed, we are only aware of a single patient achieving a pathological response to neoadjuvant immunotherapy who recurred.^55^ Spatial transcriptomic analysis of this clinical outlier demonstrated marked tumor heterogeneity,^63^ with a distinct region of immune-desert tumor, potentially mediating the early tumor relapse in this specimen. Additional studies are required to define how strong of a surrogate pathological response is in HCCs treated with neoadjuvant immunotherapy.

Despite the promising signal from these early studies, larger randomized studies are required before neoadjuvant immunotherapy may be considered a standard of care, and current guidelines indicate that neoadjuvant therapy should only be routinely performed in the context of clinical trials.^17^

### Liver transplantation

LT is the preferred strategy in patients with HCC and decompensated cirrhosis, as it offers a cure for both the HCC and the underlying liver disease. When first offered, recurrence rates were exceedingly high, and a moratorium on transplant was placed until the introduction of the Milan criteria, which facilitated a low risk of recurrence (~10% at 5 y) and prolonged OS, exceeding 60% at 5 years.^64^ There has been increased interest in expanding the benefits of liver transplant to additional patients, including proposals for expanded transplant criteria (eg, UCSF Criteria) and use of downstaging (ie, reducing tumor burden in patients beyond LT criteria to achieve eligibility). Data suggest that these patients can achieve similar survival as those who present within Milan criteria, albeit with potentially higher dropout and higher posttransplant recurrence risk. Indeed, the phase IIb/III XXL trial demonstrated improved 5 y-OS (HR: 0.32; 95% CI: 0.11, 0.92; *p* = 0·035) and 5 y-DFS (HR: 0.20; 95% CI: 0.07, 0.57; *p* = 0·003) for patients beyond Milan criteria that received LT versus those who did not.^65^

For patients who present within Milan criteria, bridging therapy is recommended to reduce the risk of tumor progression and mitigate waitlist dropout, given a 6-month wait time before being awarded MELD exception points. In patients who present beyond Milan criteria but within United Network for Organ Sharing-downstaging criteria, downstaging to within Milan criteria can be considered. Bridging and downstaging have traditionally been performed using locoregional therapies such as local ablation, TACE, transarterial radioembolization, or stereotactic body radiation therapy.^17^,^66^,^67^

**FIGURE 2:**
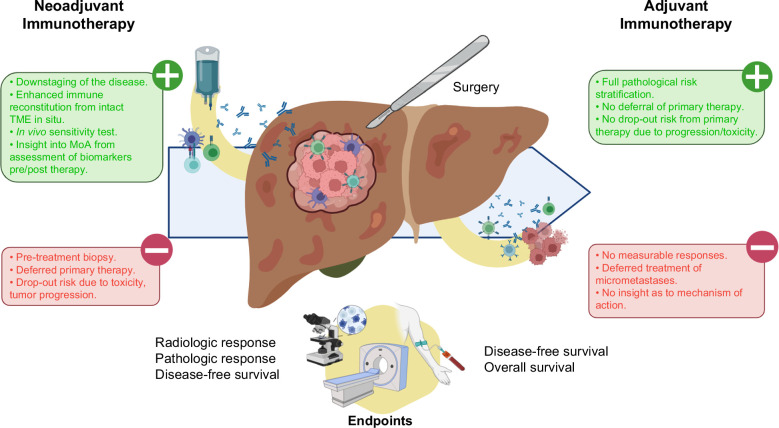
Complexities of neoadjuvant immunotherapy prior to liver transplantation. The potential benefits of immunotherapy before transplant to induce tumor responses and downstage patients to transplant must be weighed against the increased risk of allograft rejection, as these outcomes are mediated by the same mechanistic pathways of T-cell activation.

Since the introduction of sorafenib, there has been interest in investigating the role of systemic therapy for bridging or downstaging—whether alone or in combination with locoregional therapies. The rationale for combining TKI with locoregional therapy is to suppress the angiogenic impact of VEGF, which arises as a result of the hypoxic tissue conditions induced by TACE; however, most studies combining the two fail to demonstrate a benefit compared to TACE alone.^11^,^68^ Specifically, phase II and phase III studies evaluating TACE combined with brivanib (BRISK-TA), orantinib (ORIENTAL), or sorafenib (TACE-2, SPACE, TACTICS) failed to show significant improvements in time to progression or OS.^68^,^69^,^70^,^71^,^72^ The TACTICS trial comparing sorafenib + TACE versus TACE alone met its end point of progression free survival (25.2 vs. 13.5 months) but not OS. With the evolution of the systemic treatment pipeline in HCC, there are several ongoing trials evaluating the combination of immunotherapy with locoregional therapy (Table 2). However, whether immunotherapy can be safely used before or after liver transplantation remains controversial and is less clearly defined (Figure 2).

#### Use of immunotherapy as a bridge to LT

Data for the use of immunotherapy in transplant recipients were dismal from early reports with high rates of allograft rejection. An initial case series from 2014 to 2017 examined 20 recipients of transplant, including 4 recipients of liver transplant, and confirmed 50% allograft rejection with nivolumab.^73^ Similarly, data for the use of immunotherapy as bridging therapy to transplant were not much better, with a report of fatal hepatic necrosis within the first week of transplant for a patient who received nivolumab 8 days before LT.^74^ This was later challenged by a larger single-center series of 9 patients who were successfully transplanted after receiving nivolumab as neoadjuvant treatment, with 89% of patients receiving immunotherapy 4 weeks before LT. There was no reported graft loss, and only 1 patient was noted to have mild acute cellular rejection (ACR), which was successfully treated with modification of immunosuppression.^75^ A larger retrospective single-center study from China by Wang et al of 16 recipients of liver transplant who received PD1 therapy for HCC documented ACR in 9 patients (incidence of 56%, 5 received pembrolizumab) with a median time of ACR within 1 week of LT and a median FK level at the time of ACR of 7 µg/L. All patients received immunotherapy within 7–29 days from LT. Notably, all ACR episodes were reversed with treatment, and no graft or patient loss was reported due to immunotherapy.^76^ A recent literature review of all studies to date describes an allograft rejection rate of 37% (20/54 patients) with bridging therapy before LT.^77^

Toxicities from immunotherapies must be considered in this setting. Immune-related adverse events often occur early, within weeks to 3 months after initiation of treatment, although they have been documented up to 1 year after discontinuation of therapy.^78^ Among the most common immune-related adverse events include liver toxicity (5%–10% with immunotherapy monotherapy and 25%–30% with combination therapy), and because of this, prompt identification and management, including immediate discontinuation of therapy, are of paramount importance.^78^,^79^

The optimal timing for liver transplant in this setting remains unclear, but in general, stopping immunotherapy 2–3 half-lives (8–12 wk) before LT has demonstrated the lowest risk of allograft rejection and graft loss.^80^,^81^ In review of the existing literature, most reports of allograft rejection occur early, within 7–14 days after liver transplant, although there is no clear immunosuppression regimen that has been shown to prevent ACR nor was there a clear benefit to induction therapy in this setting. Close monitoring of liver function tests and immunosuppression levels in the early transplant period is critical for early identification and prompt treatment.

Although the AASLD does not recommend routine use of systemic therapies as bridging therapy prior to liver transplantation, their use does not preclude transplant eligibility.^17^ These conflicting reports bring important questions to the forefront, including which patients with HCC should be bridged with immunotherapy before liver transplant and whether there should be a required mandatory washout period. Is there a difference in outcomes with the use of Programmed cell death protein-1, PD-L1, or CTLA4 inhibitors? What is the optimal posttransplant immunosuppressive regimen? There are several ongoing trials evaluating the use of immunotherapy as bridging therapy before LT, so we should hopefully have insights into these questions in the near future.

Although careful monitoring for rejection and a washout period for immunotherapy appears to yield promising results, current data do not support its use as a standard of care for bridging therapy in clinical practice. Patients beyond United Network for Organ Sharing downstaging (including those with infiltrative disease or macrovascular invasion), markedly elevated alpha fetoprotein > 1000 ng/mL, or disease refractory to locoregional therapy are likely appropriate candidates for immunotherapy; in those patients with robust objective responses, liver transplant may be considered on a case-by-case basis.

#### Use of IO after LT

Several risk scores have been developed and validated to predict the risk of posttransplant recurrence, with one of the best-validated models being the RETREAT score.^82^ However, these models are largely used to guide surveillance strategies as there is no proven role of adjuvant therapy, even among patients at the highest risk of recurrence. Studies have reported the safety and activity of TKI-based therapy in the post-LT setting, although there are no studies examining systemic therapy in an adjuvant setting to prevent post-LT recurrence. The mainstay of management in high-risk patients remains minimization of immunosuppression, consideration of everolimus (although the data are conflicting), and surveillance monitoring to identify recurrence if/when it occurs.^83^

Recurrence of HCC after LT is associated with poor survival unless curative-intent treatments can be offered, specifically surgical resection or ablation.^84^ Although there can be a temptation to use ICI treatment for recurrent HCC or de novo cancer, systematic reviews have documented an allograft rejection rate of 25.9% and a mortality rate of 12.1%.^77^ Overall, current data highlight that posttransplant immunotherapy has high rates of ACR and graft loss, and its use is generally not recommended.

### Importance of multidisciplinary care

The complexity of HCC management, including the emergence of (neo)adjuvant therapy, highlights the importance of close collaboration between disciplines. Effective multidisciplinary care will become increasingly critical for HCC management, with the goals of confirming HCC staging, determining optimal treatments and sequences, and improving clinical outcomes. Key disciplines for multidisciplinary care of patients with HCC typically include, but are not limited to, hepatologists, radiologists, interventional radiologists, transplant and hepatobiliary surgeons, and medical and radiation oncologists.^85^ Multidisciplinary care for patients with HCC not only increases patient satisfaction but also improves timely guideline-concordant care and increases OS; highlighting this approach should be considered the standard of care for the management of patients with HCC—including for patients with early-stage HCC undergoing surgical therapies.^86^

## SUMMARY

There is a strong and growing interest in (neo)adjuvant strategies for HCC management with the recent advances in systemic therapy efficacy. The IMBrave050 Trial demonstrated improvements in RFS with adjuvant immunotherapy in high-risk patients after surgical resection or local ablation, with several other trials anticipated to be reported in the coming years. One of the key questions moving forward will be whether these therapies are better used in an adjuvant or neoadjuvant setting. Although early data suggest the latter, clinical trials are still needed to address this question.

Immunotherapy should not be considered a contraindication to liver transplantation but is currently discouraged to treat recurrence after transplant, given the high risk of rejection and graft loss. Ongoing studies are evaluating the incremental value of immunotherapy compared to locoregional therapy and addressing questions such as optimal washout period and posttransplant immunosuppression. Decisions about (neo)adjuvant therapy, including patient selection, are best made in a multidisciplinary care setting.
